# Delayed traumatic subcutaneous emphysema: a case report

**DOI:** 10.1186/s13256-025-05249-4

**Published:** 2025-05-30

**Authors:** Lucas Bishop, Sarah MacLaren, William Pollitt

**Affiliations:** 1 University of Exeter Medical School, St Luke’s Campus, Heavitree Road, Exeter, EX1 2LU UK; 2 Royal Devon and Exeter Hospital Emergency Department, Barrack Road, Exeter, EX2 5DW UK

**Keywords:** Subcutaneous emphysema, Pneumothorax, Case report, Trauma, Emergency medicine, Intensive care

## Abstract

**Background:**

Subcutaneous emphysema is a common, usually benign, and self-limiting complication of traumatic chest wall injury. In a minority of thoracic injuries, pneumothoraces can result in extensive subcutaneous emphysema and subsequent airway obstruction if air tracks along tissue planes within the neck. Furthermore, patients may have a delay to presentation following chest-wall injury and can rapidly decline. Hence, we discuss a case of delayed traumatic subcutaneous emphysema resulting in airway compromise, without cardiorespiratory compromise from tension pneumothoraces.

**Case presentation:**

A white British female in her 70s attended the emergency department 24 h after a fall at home with the complaint of right sided chest pain and shortness of breath. On arrival, the patient appeared well, with no sign of compromise. The patient rapidly deteriorated over the course of the next 30 min. Massive crepitus swelling was identified of her upper and lower limbs, head (including palpebral closure), neck, chest, and abdomen. Vocal changes and early airway obstruction features were identified. Prompt recognition of rapidly progressive subcutaneous emphysema with airway compromise, early rapid-sequence induction, chest-drain insertion, and a multidisciplinary team approach ensured a positive outcome, with discharge home after 12 days in hospital.

**Conclusion:**

Subcutaneous emphysema itself is rarely life-threatening, though it can infrequently manifest as an obstructive airway emergency. Delayed presentations are possible, and the presence of subcutaneous emphysema indicates severe chest-wall injury. Airway protection and treatment of pneumothoraces are critical interventions for these patients.

## Background

Subcutaneous emphysema is a common and usually benign condition. It often results from a pulmonary resection or penetrating and blunt thoracic trauma, with a subsequent pulmonary or tracheobronchial parenchymal rupture. Infrequently, subcutaneous emphysema can present as a life-threatening crisis with airway compromise. Additionally, traumatic subcutaneous emphysema patients can be at risk of rapid decline even days after the initial insult. Awareness of the potential for rapidly evolving subcutaneous emphysema and its prompt management in acutely unwell patients is crucial. We aim to emphasize the consequences of massive traumatic subcutaneous emphysema, the need for urgent airway protection in airway compromise, and the importance of rapid thoracic decompression; in addition, we aim to explore varying treatment modalities for subcutaneous emphysema. Hence, we describe an unusual case of blunt chest-wall trauma with bilateral pneumothoraces and airway-compromising subcutaneous emphysema, without associated cardiorespiratory compromise from tension pneumothoraces.

## Case presentation

A white British female in her 70s attended the emergency department via ambulance after falling onto her aga (a large metal oven) the previous day. She presented with right-sided chest pain and shortness of breath. At triage, her observations were normal. The ambulance crew reported a global pleural rub and wheeze. Her past medical history included asthma and hypothyroidism. Prescribed medications consisted of salbutamol and levothyroxine. The patient was a non-drinker and ex-smoker. She was previously independent with activities of daily living. The patient was given intravenous morphine and intravenous ondansetron, and then she was transferred to a major’s bay. A family member became increasingly concerned about the deterioration of the patient. The patient was urgently reviewed, identifying rapidly progressing subcutaneous emphysema. A hospital trauma call to resuscitation was made, including the general surgery team. Examination revealed rapidly progressive swelling with palpable crepitus affecting the head, neck, chest, upper limbs, and abdomen, including palpebral closure. Vocal changes and stridor were noted. Initial observations in the resuscitation room demonstrated an oxygen saturation of 90% on high flow oxygen but were otherwise within normal limits. 

## Investigations

### Laboratory results

- Arterial blood gas (ABG) results postintubation indicated a mixed respiratory and metabolic acidosis (pH 7.17, pO_2_ 25 kPa, pCO_2_ 8.4 kPa, and HCO_3_ 19.9 mmol/L).
- Full blood count indicated a raised white cell count (14.4 10^9^/L) and neutrophil count (11.36 10^9^/L) but normal red blood cell studies.
- Renal function tests identified hyponatremia (130 mmol/L).
- Coagulation studies (including Clauss fibrinogen) were normal.

### Imaging (all original)

See Fig. 1

**Fig. 1:**
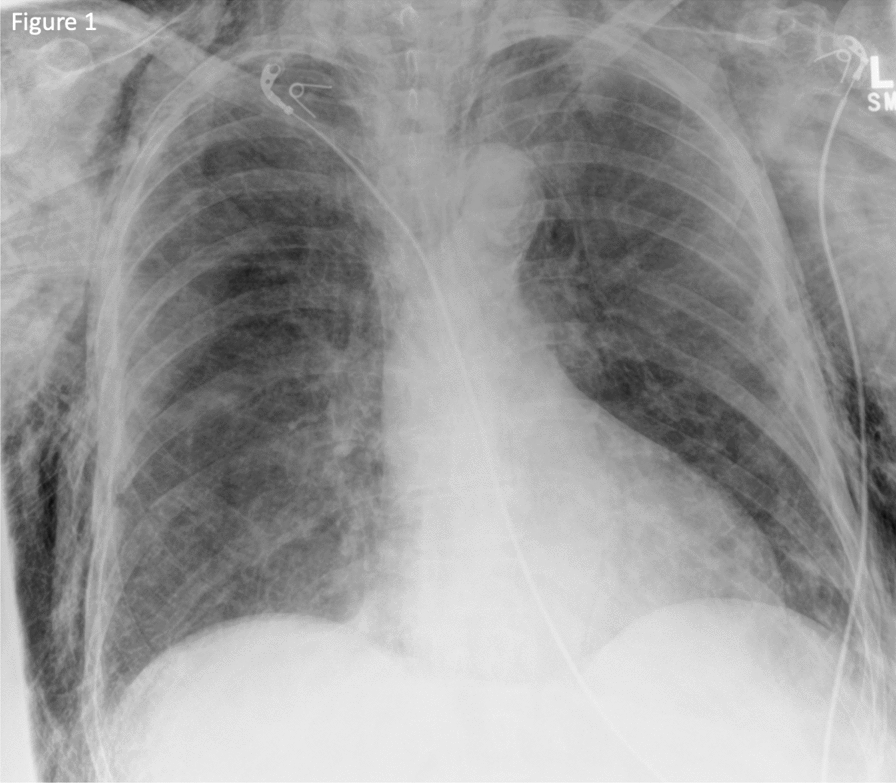
An anteroposterior portable chest X-ray taken in the resuscitation room post-intubation, demonstrating severe subcutaneous emphysema. Pneumothoraces and are not appreciable in this image, due to overlying emphysema

### Computed tomography (CT) trauma

### Management

**Fig. 2:**
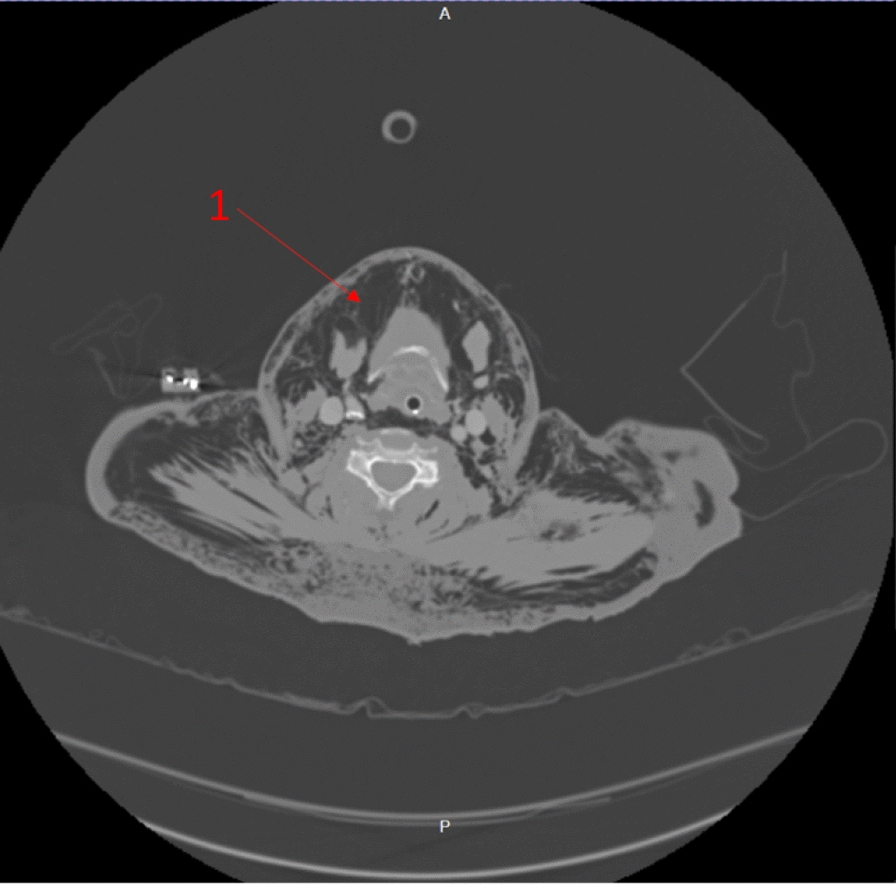
Axial computed tomography showing emphysema surrounding the larynx and laryngeal edema (arrow 1)

**Fig. 3:**
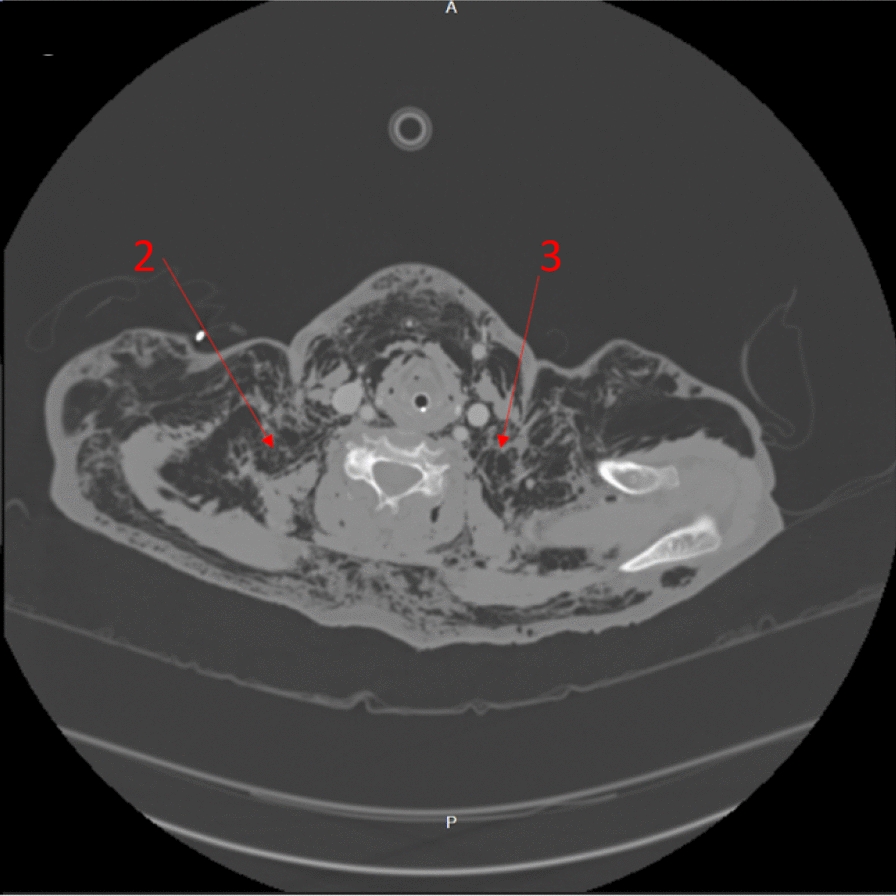
Axial computed tomography showing emphysema penetrating the deep neck tissues (arrows 2 and 3)

**Fig. 4:**
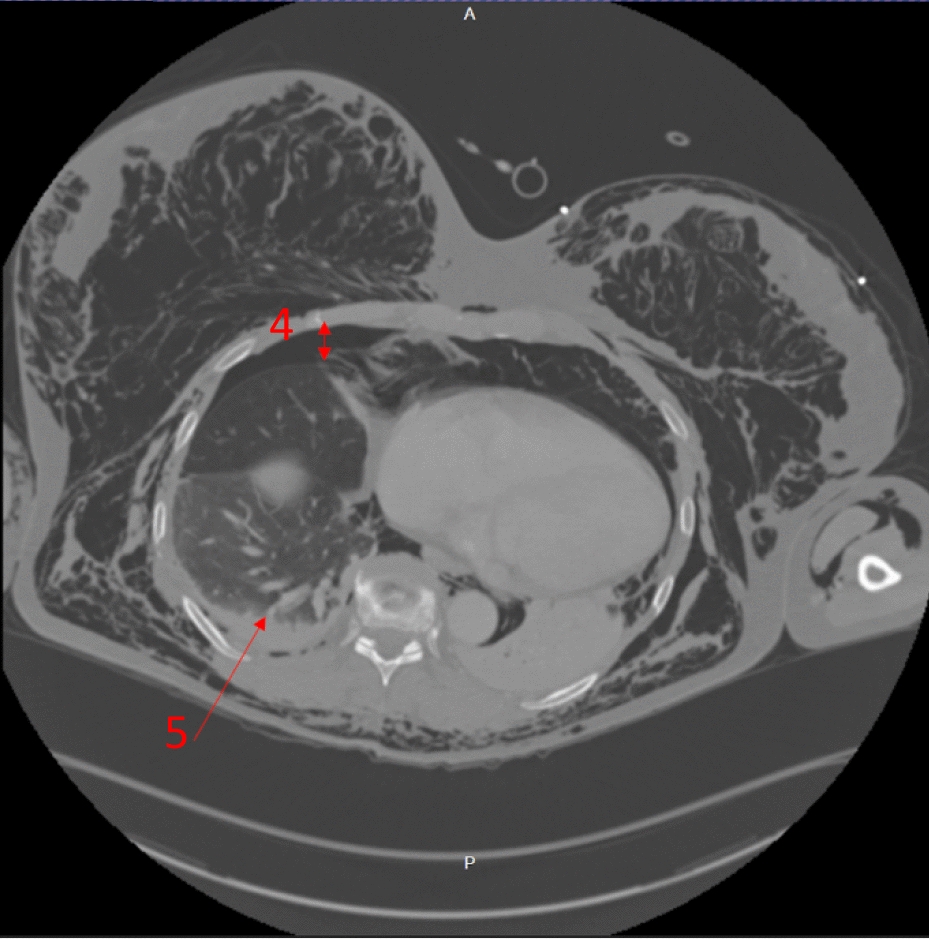
Axial computed tomography showing the right-sided moderate-volume pneumothorax (arrow 4) and the right-sided lower-lobe pulmonary hemorrhage (arrow 5)

**Fig. 5:**
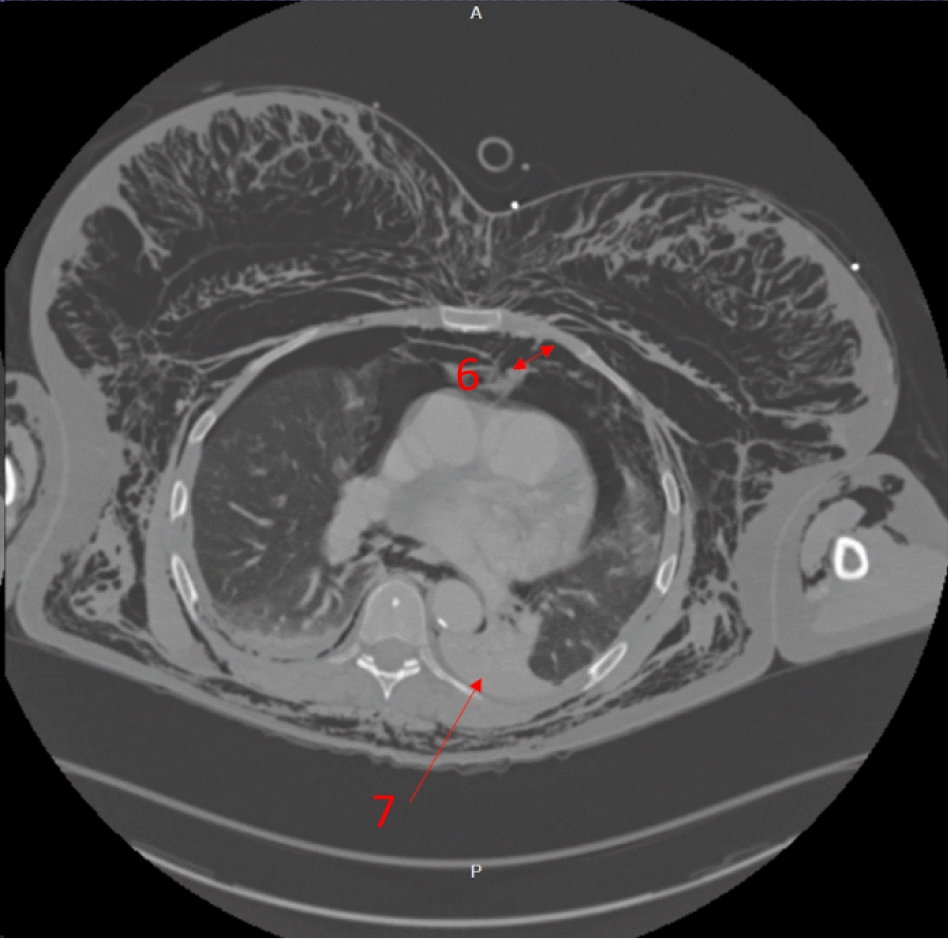
Axial computed tomography showing the left-sided moderate-volume pneumothorax (arrow 6) and the left-sided lower-lobe laceration and collapse (arrow 7)

The immediate concern in this patient was airway compromise and impending obstruction. The trauma team prioritized securing the airway, balancing potential consequences of positive pressure ventilation converting simple pneumothoraces into tension pneumothoraces. The trauma team were prepared to perform bilateral open chest drains simultaneously to rapid-sequence induction (RSI) should the patient become unstable, suggesting that any pneumothoraces had tensioned. However, the contemporaneous clinical impression based on clinical findings and imaging was that there was not significant tension of pneumothoraces, with airway compromise being the immediate threat to life, and management was not to be delayed addressing pneumothoraces. RSI was commenced with ketamine, rocuronium, and fentanyl. The patient was intubated with a C-mac video laryngoscope, a grade-1 view was identified, and a bougie was used to pass the endotracheal tube. Airway anatomy was noted as highly edematous by the intubating clinician. High respiratory pressures were present with the patient lying flat. The patient was ventilated with a tidal volume of 350 ml and respiratory rate of 15 to minimize airway pressure and reduce risk of causing tension. Anesthesia was maintained with a propofol infusion. The patient tolerated the anesthetic well, with no periods of hypotension or other adverse effects. A chest radiograph was taken in resuscitation postintubation (Fig. 1). Owing to stability post-RSI, a CT scan was obtained prior to chest-drain insertion with the rationale of obtaining further information on the underlying injury. The team were prepared for immediate decompression and drain insertion throughout time in the CT scanner. CT imaging identified extensive thoracic injuries (Figs. 2, 3, 4, 5). Fractures of ribs 7, 8, and 9 in two places on the right side were also reported. The multiple fracture in rib 9 did not present clinically as a flail chest. On immediate return, bilateral 28Fr drains were inserted in the emergency department, initially draining air. The procedure was technically challenging, with 10–15 cm of edematous tissue overlying the ribs. A second operator was required to hold the breast tissue away from the insertion site. The procedures were performed with no complications. A commiserate reduction in ventilatory pressures was noted following the insertion of the chest drains.

In the intensive care unit, the patient remained ventilated and sedated for 3 days. Initial sedation consisted of 1% propofol at 15 ml/h and alfentanil at 4 ml/h, requiring metaraminol support at 2.5 ml/h. Heavy sedation was stopped on day 2 with the aim of extubation, though the patient struggled with severe pain resulting from the rib fractures, and further light sedation with remifentanil at 6.3 ml/h was required. With successful extubation, the patient’s chest drains were removed, and there were no reported complications. A right-sided erector spinae block was performed with good effect for pain relief. Postextubation pain remained a significant obstacle for this patient, requiring several approaches including diclofenac, ibuprofen, tramadol, and, eventually, a ketamine infusion. The hospital for which this patient was admitted does not have a cardiothoracic department on site, and typically manages chest-wall trauma patients who do not require surgical rib fixation on a general surgical ward with an established thoracic trauma pathway. The regional cardiothoracic service can be contacted for advice and acceptance of patients when they are deemed to require tertiary care. This woman had fractures of the seventh, eighth, and ninth ribs reported, with the ninth being fractured in 2 places, but without a clinical flail chest. Hence, the decision was made for nonoperative management.

On day 7, the patient was stepped down to a surgical ward under the established chest-wall trauma pathway of the treating hospital. Pain management consisted of tramadol and oxycodone. Salbutamol nebulizers for her asthma were utilized on the ward. The patient was discharged home on day 12, with lidocaine patches, oxycodone, and tramadol for analgesia.

## Outcome and follow-up

A total of 1 month after discharge, the patient was reviewed by physiotherapy. Recovery for this patient has been good. She has been able to begin undertaking her usual activities. Though these functions have returned, the patient did describe difficulties with tiredness and generalized weakness. Several months after initial follow-up, the patient reported similar troubles, but progressive improvement.

## Discussion

Subcutaneous emphysema is an infrequent and most often benign condition that arises through several processes including barotrauma, blunt and penetrating injuries, infection, and surgical complications [1, 2]. Subcutaneous emphysema is most commonly associated with pneumothoraces [1]. Underlying pathophysiology of subcutaneous emphysema follows several routes: rupture of pulmonary or tracheobronchial parenchyma allowing airflow into subcutaneous tissue and along fascial planes, mediastinal air spreading to cervical viscera, air from external sources, and local gas generation from infection [3]. Early clinical signs include swelling and crepitus of the affected area. Even in stable patients, palpebral closure and many other sequelae of subcutaneous emphysema can be exceptionally uncomfortable and distressing. Additional complications of subcutaneous emphysema include blinding, compartment syndrome, intracranial hypertension, and pacemaker malfunction [2].

Deterioration in traumatic subcutaneous emphysema patients most often occurs secondary to concurrent tension pneumothoraces, resulting in obstructive shock and respiratory failure. Case reports demonstrate a minority of patients with massive subcutaneous emphysema can present acutely with airway obstruction from air infiltration into the cervical fascial planes and laryngeal edema [4–8]. Importantly, the presence of subcutaneous emphysema has been identified as a significant predictor of severe chest-wall injury regardless of severity, and clinicians should be cognizant of the risk of rapid decline [9, 10]. Additionally, the onset of tension pneumothoraces and subcutaneous emphysema in thoracic trauma may be delayed even days after the initial insult [7, 8, 11–13]. Underlying mechanisms behind these delayed presentations are thought to relate to incidences of raised intrathoracic pressure worsening or inducing lung parenchymal injury through straining, coughing, or the Valsalva maneuver [9, 12]. This patient’s complications were likely precipitated by coughing, leading to pulmonary parenchymal injury from penetrating rib fractures and consequent bilateral pneumothoraces. This was then complicated with the development of massive subcutaneous emphysema and airway obstruction.

Identifying the source of subcutaneous emphysema is of priority in initial investigation in chest trauma. Chest radiographs lack the sensitivity to identify small pneumothoraces, as demonstrated in this case (Fig. 1), with computed tomography (CT) providing the best diagnostic capabilities [10, 14]. Most pneumothoraces with associated subcutaneous emphysema should be addressed through a chest-drain insertion, with a conservative approach being taken on the subcutaneous emphysema itself [2, 14]. In the presence of subcutaneous emphysema-induced airway obstruction, the immediate priority is to establish airway control. The majority of subcutaneous emphysema cases are self-limiting and will resolve with time [2]. Refractory or symptomatic subcutaneous emphysema can be managed through various techniques such as subcutaneous drains, skin incisions, subcutaneous angio-catheters, massage techniques, and vacuum-assisted closure (VAC) therapy [2, 15, 16]. The efficacy of each technique varies, though all are thought to yield better outcomes than no active intervention [2, 16]. Moreover, all of these are able to provide rapid relief and comfort to the patient with minimal reported complications [2, 15, 16]. It has been hypothesized that several cohorts of patients with pneumothoraces and subcutaneous emphysema should receive high flow oxygen regardless of their oxygen saturations [2, 17]. Supplying oxygen aids in the correction of hypoxemia when needed and also increases the oxygen content of air within pneumothoraces and emphysematous subcutaneous tissue. This air is then potentially reabsorbed from the pleural cavity and subcutaneous tissue at a greater rate owing to the potent diffusion capabilities of oxygen [2, 14]. In turn, this can potentially provide a noninvasive adjunct to increase the rate of resolution for both pneumothoraces and subcutaneous emphysema.

## Conclusion

Traumatic subcutaneous emphysema is a common and often benign complication of chest trauma. In a minority of cases, subcutaneous emphysema can evolve to produce airway obstruction. Delayed presentations are possible within this cohort, and clinicians should be mindful of the presence of subcutaneous emphysema indicating severe chest-wall injury. Definitive airway protection in those with obstruction and rapid correction of tension pneumothoraces are the pillars of life-saving intervention. Clinician awareness of varying treatment modalities for subcutaneous emphysema can provide both symptomatic relief and improve patient outcomes.

## Data Availability

Data sharing is not applicable to this article as no datasets were generated or analyzed during the current study.
